# Integrated metabolomics and proteomics reveal biomarkers associated with hemodialysis in end-stage kidney disease

**DOI:** 10.3389/fphar.2023.1243505

**Published:** 2023-11-27

**Authors:** Weiwei Lin, Fatemeh Mousavi, Benjamin C. Blum, Christian F. Heckendorf, Jarrod Moore, Noah Lampl, Mark McComb, Sergei Kotelnikov, Wenqing Yin, Nabil Rabhi, Matthew D. Layne, Dima Kozakov, Vipul C. Chitalia, Andrew Emili

**Affiliations:** ^1^ Center for Network Systems Biology, Boston University, Boston, MA, United States; ^2^ Department of Biochemistry, Boston University School of Medicine, Boston, MA, United States; ^3^ Department of Applied Mathematics and Statistics, Stony Brook University, Stony Brook, NY, United States; ^4^ Renal Section, Department of Medicine, Boston University School of Medicine, Boston, MA, United States; ^5^ Veterans Affairs Boston Healthcare System, Boston, MA, United States; ^6^ Institute of Medical Engineering and Sciences, Massachusetts Institute of Technology, Cambridge, MA, United States; ^7^ Department of Biology, Boston University, Boston, MA, United States

**Keywords:** metabolomics, proteomics, nLC-MS/MS, ESKD, integrated omics

## Abstract

**Background:** We hypothesize that the poor survival outcomes of end-stage kidney disease (ESKD) patients undergoing hemodialysis are associated with a low filtering efficiency and selectivity. The current gold standard criteria using single or several markers show an inability to predict or disclose the treatment effect and disease progression accurately.

**Methods:** We performed an integrated mass spectrometry-based metabolomic and proteomic workflow capable of detecting and quantifying circulating small molecules and proteins in the serum of ESKD patients. Markers linked to cardiovascular disease (CVD) were validated on human induced pluripotent stem cell (iPSC)-derived cardiomyocytes.

**Results:** We identified dozens of elevated molecules in the serum of patients compared with healthy controls. Surprisingly, many metabolites, including lipids, remained at an elevated blood concentration despite dialysis. These molecules and their associated physical interaction networks are correlated with clinical complications in chronic kidney disease. This study confirmed two uremic toxins associated with CVD, a major risk for patients with ESKD.

**Conclusion:** The retained molecules and metabolite–protein interaction network address a knowledge gap of candidate uremic toxins associated with clinical complications in patients undergoing dialysis, providing mechanistic insights and potential drug discovery strategies for ESKD.

## Introduction

End-stage kidney disease (ESKD) is a form of advanced chronic kidney disease (CKD) wherein the renal filtering capacity becomes insufficient to remove circulating metabolic waste and excess fluid from patient’s blood. This results in the accumulation of uremic toxins, a life-threatening condition that necessitates kidney transplant or dialysis. Since kidney replacement is challenging, hemodialysis is widely applied to ESKD patients. During this procedure, blood is pumped through a dialyzer with a membrane designed to filter out toxins to restore fluid and electrolyte balance (Mohajerani et al., 2022). Although not a replacement for kidney function, dialysis remains a life-saving option for patients and the current standard-of-care therapy for patients with ESKD. However, the mortality remains high. Poor clinical outcomes are associated with an elevated risk of infection, anemia, and cardiovascular disease (CVD) (Virani et al., 2008). The mechanisms driving these outcomes are not fully understood, but they reflect the cumulative complexity of disease progression, the side effects of dialysis, and potential comorbidities (*e.g*., hypertension and diabetes) (Bello et al., 2017). Blood creatinine levels and urine output are commonly used as clinical markers of disease progression in CKD, but they show limited accuracy for predicting future complications in ESKD patients undergoing hemodialysis.

Liquid chromatography coupled to tandem mass spectrometry (LC/MS) enables sensitive, high-resolution, large-scale identification of circulating biomolecules (Dubin and Rhee, 2020). In principle, LC/MS-based proteomic and metabolomic profiles can provide complementary insights into the biochemical pathways altered during ESKD, revealing potential prognostic markers that could be used to improve clinical outcomes. Although a host of uremic toxins have been defined as cardiovascular risk factors, there is a dearth in studies comprehensively evaluating compounds that are preferentially retained at elevated levels in the plasma of patients undergoing hemodialysis. This knowledge gap compromises the mechanistic understanding and treatment of CVDs and other complications in patients with ESKD (Ravid et al., 2021). Although previous studies have documented a small set of candidate uremic toxins that are not effectively removed by hemodialysis, we hypothesized that the persistence of additional factors impacts clinical outcomes.

By combining robust nanoflow metabolomic and proteomic workflows, our study has gained a more comprehensive picture of the molecular changes and associated biochemical pathways and physical interaction networks that remain altered in ESKD disease. Our integrated analysis identified dozens of candidate uremic toxins, 21 of which were independently further confirmed as persistently elevated in patient plasma using reference standards. Additionally, we uncovered multiple factors with potential links to CVD, a major determinant of mortality among patients with ESKD (Himmelfarb et al., 2020).

## Materials and methods

### Chemical and materials

LC-MS grade solvents were obtained from Fisher Scientific. The solid-phase micro-extraction (SPME) blade unit and robotic 96 auto-sampler were purchased from Professional Analytical Systems Technology (Magdala, Germany) for metabolomic crude extracts’ clean-up. Metabolite standards with purity greater than 95% were purchased from MetaSci (Canada). Doxycycline was purchased from Sigma. DMSO (Sigma) was used as a vehicle control.

### Clinical samples

The ESKD serum samples were collected from patients at the Renal Section of the Department of Medicine at the Boston Medical Center. The protocols for patient recruitment and sample collection at Boston University Medical Campus were IRB-approved (H-26367) and supervised by Dr. Vipul Chitalia. Control serum (healthy cohort) was obtained from Research Blood Components, LLC (MA). All participants provided informed consent for the use of their blood for research purposes. Whole blood samples (∼1 mL) from both the healthy cohort and ESKD patients (both before and after hemodialysis procedure) were collected and allowed to sit for 30 min at room temperature, and then centrifuged at 3,000 rpm at 4°C for 10 min. Serum (supernatant) was transferred to new tubes and quenched immediately at −80°C prior to metabolite extraction.

### Human induced pluripotent stem cell-derived cardiomyocyte cell culture and cytotoxicity assay

Human induced pluripotent stem cell (hiPSC)-derived cardiomyocytes were generously provided by the Seidman Lab at Harvard Medical School. In brief, hiPSCs were cultured with mTESR1 media (STEMCELL Technologies) and differentiated into cardiomyocytes (day 0) via activation of the WNT pathway with 12 μM CHIR 99021 (Tocris) in RPMI + GlutaMAX media supplemented with B27 minus insulin (RPMI and B27 minus, Thermo Fisher Scientific). After 48 h, the WNT pathway was inhibited via 5 μM IWP-4 (Tocris). On day 9, the media was replaced with RPMI + GlutaMAX media supplemented with B27 plus insulin (Thermo Fisher Scientific). On days 11 and 13, the cardiomyocyte population was purified by metabolic selection via RPMI glucose-free media (Gibco) supplemented with 4 mM of DL-lactate (Millipore Sigma). On day 15, cardiomyocytes were placed in 50% FBS in PBS to deplete the protease, centrifuged, and the supernatant was aspirated. They were then placed in RPMI B27+ supplemented with 5 μM Y-27632 (Tocris) and 2% fetal bovine serum (MilliporeSigma) for seeding.

Homocysteine, taurine, and positive control (doxorubicin) were dissolved into 1 mM stock solution using 0.5% DMSO and then serially diluted to a desired final concentration (100 μM, 10 μM, 1 μM, 100 nM, and 10 nM) in media prior to cell treatment. The toxicity of vehicle (0.5% DMSO) alone was evaluated and found not to cause any noticeable toxicity on iPSC-derived cardiomyocytes. For the cytotoxicity assay, the cells were incubated with their respective metabolites for 24 h at 37°C and 5% CO_2_ in five replicates. Then, the cells were incubated with a 10 μL cell proliferation reagent WST-1 (Sigma-Aldrich) for 4 h at 37°C and 5% CO_2_. After shaking for 1 min, the samples were read on a microplate reader at 420 nm absorbance.

### Metabolite extraction

Patient serum (50 μL) was resuspended in a 4 vol. mixture of ice-cold methanol/acetonitrile/water (MeOH/ACN/H_2_O, 40/40/20 v/v; Fisher) in chemically resistant microcentrifuge tubes (*e.g.,* Eppendorf) and vortexed for 30 s. The mixture was incubated for 1h at −20°C and centrifuged at 12,000 x g and 4°C for 15 min to pellet the protein precipitate. The metabolite-containing supernatants were transferred to new tubes, dried under vacuum at 30°C, and kept at −80°C prior to LC-MS. For LC-MS analysis, extracts were thawed and resolubilized in 200 μL of 2% methanol and subjected to SPME (see the following section) for sample clean-up. The protein precipitate was maintained at 4°C prior to tryptic digestion and proteomic analysis. Equal amounts of each sample were pooled as an internal quality control (QC).

### Solid-phase micro-extraction

The metabolite mixtures were transferred to a 96-well plate for SPME processing. The coated blades were washed with EtOH/H_2_O (70:30, v/v) for 30 min and preconditioned for 30 min in MeOH/H_2_O (50:50, v/v). The samples were extracted by incubation with the blades for 1 h with shaking. The blades were briefly rinsed for ∼20 s using water, and then, the bound metabolites were desorbed using ACN/H_2_O (50:50, v/v) for 1 h. The solvent was evaporated to dryness using a vacuum concentrator at 30°C. For LC-MS analysis, metabolites were reconstituted in 20 μL of 2% ACN. To prevent carryover particles, metabolites were centrifuged at 10,000 x g at 4°C for 15 min, the supernatant was transferred to a new tube, and this step was repeated one more time prior to injection.

### LC-MS analysis of metabolites

The analysis was performed on an Orbitrap Exploris 480 mass spectrometer (Thermo Fisher Scientific) interfaced to the EASY nanoLC1200 system (Thermo Fisher Scientific). The metabolites (including lipids) were loaded onto a C18 reverse-phase pre-column (75 μm i. d. × 2 cm, 3 μm) and then separated by a capillary column (75 μm i. d. × 25 cm, 2 μm, 100 Å, Thermo Fisher Scientific); the column oven was set to 40°C. The mobile phase A was 2% ACN, and the mobile phase B was 80% ACN. The nLC flow rate was 300 nL/min. The samples (3 μL) were injected and separated over a 45-min gradient. The gradient consisted of 2%–60% mobile phase B for 20 min, was increased to 95% mobile phase B over 10 min, and maintained at 95% mobile phase B for 15 min. The MS instrument was operated in the automated switching ESI mode over a full mass scan range of m/z 67–1,000 at a resolution of 60,000. The AGC target was set to 300% (equal to 3×e^6^ ions), and the maximum ion injection time was set to 25 ms. The source ionization parameters were optimized for a transfer temperature at 300°C, and a spray voltage was set to 2.1 kV and −1.8 kV for the positive and negative modes, respectively. MS2 scans were performed at 15,000 resolution with a maximum injection time of 64 ms using stepped normalized collision energies (NCEs) of 10, 20, and 40. Dynamic exclusion was enabled using a time window of 10 s.

### Protein digestion

Five replicates were randomly selected from each condition for the pooled (multiplex) proteomic analysis. After pelleting, the protein precipitates from the organic solvent extraction were resuspended in 250 μL of lysis buffer containing 6 M guanidine hydrochloride (GuHCl), protease inhibitors (Sigma), and phosphatase inhibitors (Roche). The samples were heated at 95°C for 10 min, cooled on ice for 10 min, and then briefly sonicated to shear the nucleic acids. The samples were diluted with 100 mM Tris (pH 8.5) to reduce the concentration of GuHCl to 0.75 M. After quantification with a BCA kit (Thermo Scientific), the proteins were digested overnight with sequence-grade trypsin (enzyme-to-protein ratio of 1:50) at 37°C, and formic acid was then added to obtain a final concentration of 1% in solution. The resulting peptides were desalted using a C18 Sep-Pak cartridge (Waters), according to the manufacturer’s instructions.

### TMT peptide labeling

Prior to tandem mass tag (TMT) labeling, peptide quantification was performed by the Pierce quantitative colorimetric assay (Thermo Scientific). According to the manufacturer’s instructions, 100 μg of peptide per sample was resuspended in 0.1 M triethylammonium bicarbonate (TEAB). Peptides (five channels per condition, healthy, and pre- and post-dialysis) were labeled with TMTpro (Thermo Scientific) for 1 h at room temperature. To quench the reaction, 5% hydroxylamine was added to each sample, and the resulting mixture was incubated at room temperature for 15 min. After labeling, equal amounts of each sample were combined in a new microtube and desalted using a C18 Sep-Pak cartridge (Waters).

### High-pH reverse-phase peptide fractionation

Peptides (500 μg) were fractionated offline on a Waters XBridge BEH C18 reverse-phase column (3.5 μm, 4.6 × 250 mm) using an Agilent 1100 HPLC system operated at a flow rate of 0.45 mL/min with two buffer lines: buffer A (consisting of 0.1% ammonium hydroxide–2% acetonitrile–water) and buffer B (consisting of 0.1% ammonium hydroxide–98% acetonitrile, pH 9). The peptides were separated by a gradient from 0% to 10% B in 5 min, followed by linear increases to 30% B in 23 min, to 60% B in 7 min, and then 100% in 8 min and maintained at 100% for 5 min. This separation yielded 48 collected fractions that were subsequently combined into 12 fractions and then evaporated to dryness in a vacuum concentrator. The peptides (2 µg) from each fraction were reconstituted in 0.1% formic acid and maintained at −80°C prior to analysis by nLC-MS/MS.

### LC-MS analysis of peptides

An Orbitrap Exploris 480 mass spectrometer, interfaced with an EASY nanoLC1200 ultra-high pressure pump system, was used for peptide analysis. Peptides were loaded onto a C18 pre-column (75 μm i. d. × 2 cm, 100 Å, Thermo Fisher Scientific) and then separated on a reverse-phase nano-spray column (75 μm i. d. × 50 cm, 100 Å, Thermo Fisher Scientific) over a 150-min gradient. Mobile phase A consisted of 0.1% FA-2% ACN–water, and mobile phase B consisted of 0.1% FA-80% ACN–water. The gradient consisted of 6%–40% mobile phase B over 155 min, was increased to 95% mobile phase B over 4 min, and maintained at 95% mobile phase B for 3 min at a flow rate of 250 nL/min. The MS instrument was operated in a positive ion mode over a full mass scan range of m/z 350–1,400 at a resolution of 60,000 with a normalized AGC target of 300%. The source ion transfer tube temperature was set at 275°C, and a spray voltage was set to 2.5 kv. Data were acquired on a data-dependent mode with FAIMS running three compensation voltages at −50v, −57v, and −64v. MS2 scans were performed at 45,000 resolution with a normalized collision energy of 34. Dynamic exclusion was enabled using a time window of 60 s.

### Metabolomic data processing

The raw chromatographic data files were converted to the mzML format and split into positive and negative ion mode files with msConvert (Adusumilli and Mallick, 2017) prior to analysis using MS-DIAL (V4.18) (Tsugawa et al., 2015). ‘Linear-weighted moving average’ was used for peak detection. The minimum peak height was set to 20,000. Afterward, spectral centroiding was performed by integrating the mass spectrum over the ±0.01 and ±0.025 Da ranges in MS1 and MS2, respectively. The spectra were searched using MS-DIAL (Tsugawa et al., 2015) against a metabolomic (MSMS-Public-Pos-VS15. msp) or lipidomic (LipidMsmsBinaryDB-VS68-FiehnO.lbm2) library with a matched mass tolerance of 0.025 and 0.05 Da for MS1 and MS2 ions, respectively. The QC samples were specified as reference files for sample alignment. The data matrixes were exported as tab-delimited text files. Features with high CV intensity (≥50), low fold change (≤5) in average sample intensity relative to negative controls, low signal/noise (≤3), and peak widths less than 6 scans were removed prior to subsequent downstream analysis. The accurate mass, retention time, and MS/MS spectra of metabolite standards were extracted and used to confirm the putative metabolites detected in ESKD, limiting by mass and retention time shift and validating by spectral similarity (matching score >0.7).

We confirmed 21 altered candidate metabolites (*p* < 0.05, ≥1.5-fold-change in ESKD *versus* control), by matching accurate mass, retention time, and/or MS2 spectrum with reference standards (MetaSci, Canada, see Table 2 for identifications). Compounds (purity≥95%) that were dissolved in ∼50% acetonitrile, methanol, iso-propanol, or chloroform could be used for water-insoluble compounds. Pooled metabolite standards (∼100 ng/mL) were separated using the same C18 pre-column and EASY-Spray column, and the same method was applied to acquire MS1 and MS/MS spectra. Metabolites were identified by accurate mass (≤1ppm for the positive mode;≤2ppm for the negative mode), retention time, and MS2 similarity (matching score≥0.7).

### Proteomic data analysis

MS2 spectra were processed and searched by MaxQuant (version 1.6.7) against the UniProt FASTA database (uniprot.org) downloaded on 2020-02-10 containing all canonical reviewed human protein sequences from Swiss-Prot. The search allowed for two missed trypsin cleavage sites, variable modifications of methionine oxidation, and N-terminal acetylation. The carbamidomethylation of cysteine residues was set as a fixed modification. Ion tolerances of 20 and 6 ppm were set for the first and second searches, respectively. Protein and peptide identifications were filtered at a 1% FDR threshold based on searching the target-decoy database strategy (Elias and Gygi, 2007). The candidate peptide identifications were filtered assuming a 1% FDR threshold based on searching the reverse sequence database. Quantification was performed using the TMT reporter on MS2. The reporter ion intensities were log-transferred and normalized based on quantiles. Protein–lipid 3D structure modeling was performed using an in-house template-based LigTBM protein–ligand docking protocol (Alekseenko et al., 2020). Bioinformatic analysis was performed in the R statistical computing environment (version 3.6.1).

### Metabolite–protein network analysis

Enrichment analysis and disease-associated pathway analysis were performed against the set of metabolites elevated in ESKD using the web-based tool MetaboAnalyst 5.0 (Chong and Xia, 2020) to search the Small Molecule Pathway Database (SMPDB, https://www.smpdb.ca/). Metabolites resulting from the combination of category I and category II represented the significantly elevated compounds in ESKD compared with control, regardless of dialysis, while category II alone represented the persistent toxins; category III (down in ESKD) consisted of few metabolites. Analysis of these three groups of metabolites revealed both the changed metabolomic patterns of ESKD and, more notably, the (incomplete) effects of dialysis. We performed joint protein–metabolite network analysis based on significantly elevated metabolites (category I + category II annotations) and differential proteins (FC > 1.5, *p* < 0.05, including both up and downregulation) detected in patient serum samples. Metabolite and protein (enzyme/gene) associations were extracted from STITCH (Kuhn et al., 2008), a database including interaction networks of small molecules and proteins based on curated biochemical reactions from a similar chemical structure and similar molecular activities. The resulting association networks, consisting of physically associated metabolites and proteins and their direct neighbors, were visualized using Cytoscape 3.9.1.

## Results

### Metabolomic and proteomic profiles differ during hemodialysis

Our unified workflow detects metabolite, lipid, and protein levels in serum samples collected from a matched cohort of ESKD patients (n = 10) before and after hemodialysis along with the healthy cohort (n = 10) (see Table 1 for patient characteristics). Renal function was estimated by the glomerular filtration rate (eGFR). ESKD patients (George and Gounden, 2019) had a significantly lower eGFR (mean = 8.1 ± 7.89 mL/min/sq mt body) than the age- and sex-matched cohort (>90 mL/min/sq mt body) (Table 1). This cohort consisted primarily of African American and non-White Hispanic patients, with approximately half being male patients. All of the patients presented at least one comorbidity, including diabetes (80%), CVD (50%), peripheral artery disease (20%), and a remote history of deep vein thrombosis (60%) and pulmonary embolism (20%) (Table 1).

**TABLE 1:** Patient characteristics.

Study/baseline participant	Patients with ESKD on HD	Control
	N = 10	N = 10
Age, year#	Mean: 64.4 ± 10.22	Mean: 50.2 ± 8.61
Male	5 (50%)	6 (60%)
BMI #	Mean: 31.63 ± 5.39	Mean: 25.72 ± 7.25
Ethnicity		
Hispanic	2	6
Non-Hispanic	8	4
Race		
Black population	8	4
White population	2	6
Asians	0	0
American Indians	0	0
Others	0	0
Smoke	6	0
Hypertension	10	0
Diabetes mellitus	8	0
Cardiovascular diseases		
Documented CAD	5	0
Angina	6	0
Coronary artery bypass	5	0
Congestive heart failure	5	0
Hypercholesterolemia	5	0
Peripheral vascular disease	2	0
Deep venous thrombosis	6	0
Pulmonary embolism	2	0
Blood pressure, mmHg		
Systolic#	Mean: 140.7 ± 22.31	Mean: 125 ± 14.69
Diastolic#	Mean: 79.2 ± 15.89	Mean: 74 ± 6.53
eGFR ml/min/sq mt body	Mean: 8.1 ± 7.89	Mean: >90

**FIGURE 1:**
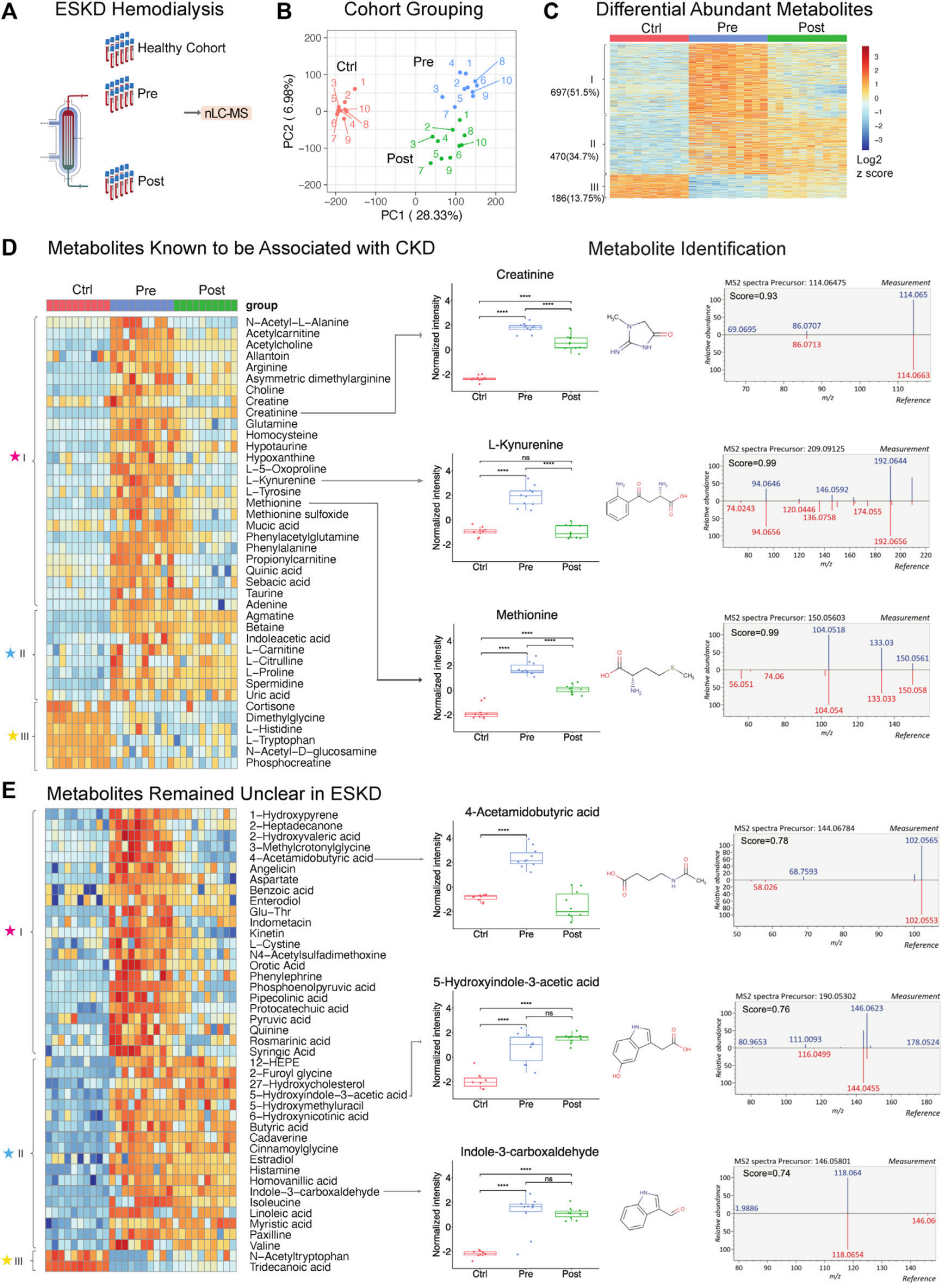
Untargeted metabolomic profiling of serum from patients with end-stage kidney disease. **(A)** ESKD clinical sample collection and processing. **(B)** PCA plot of LLE-SPME-nLC/MS sample profiles (healthy CTRL controls vs*.* pre- and post-dialysis ESKD cohorts). **(C)** Heatmap of differentially abundant metabolites (*p* < 0.05, >2-fold change (FC), FDR<0.05); metabolites are classified as class I (increased pre-dialysis, decreased post-dialysis), class II (increased pre-dialysis, but not significantly reduced post-dialysis), and class III (decreased in ESKD). **(D)** Heatmap of uremic solutes associated with CKD along with representative experimental data showing the circulating levels (relative intensity) and matched MS/MS fragmentation patterns obtained for creatine, L-kynurenine, and methionine (chemical structures from HMDB: https://hmdb.ca/). **(E)** Heatmap of metabolites significantly elevated (>5-fold change, *p* < 0.05, FDR<0.05) in EKSD along with representative data obtained for 4-acetamidobutyric acid, 5-hydroxyindole-3-acetic acid, and indole-3-carboxaldehyde.

Serum samples were collected both pre- and post-dialysis (schematic representation in Figure 1A) and compared with serum from healthy controls matched for age, sex, and body mass index without diagnostic comorbidities (Table 1). The samples (three groups, n = 30 in total) were quenched by quick freezing and processed using a unified technical workflow to acquire metabolomic and lipidomic (in the same LC/MS run) data and proteomic data from the same biospecimens. In brief, protein was precipitated using cold MeOH/ACN/H_2_O (4/4/2, v/v), while soluble metabolites and lipids were collected from the supernatant by SPME, an in-house sample clean-up approach using resins optimized to selectively retain both polar and non-polar metabolites while removing matrix contaminants.

A total of 8,410 putative metabolites (both positive and negative modes) and 665 proteins (without depletion of albumin or other high-abundance blood proteins) were detected in serum (Supplementary Table S1; Supplementary Table S2). Strikingly, dozens of metabolites, lipids, and proteins that accumulated in the ESKD patient serum remained at high levels relative to healthy controls even right after hemodialysis.

### Persistently elevated metabolites in ESKD patients undergoing hemodialysis

PCA plots revealed the distinct metabolomic profiles of each sample cohort (Figure 1B, C). Notably, 1,167 putative metabolites were significantly elevated (*p* < 0.05, FDR<0.05, ≥2-fold change; Supplementary Table S1) in the serum from patients with ESKD before dialysis (Pre, *blue*) relative to controls (Ctrl, *red*) (Figure 1C), while only 186 metabolites were decreased**.** These included 697 metabolites significantly decreased after dialysis and 470 of them showed no significant decrease after dialysis compared with pre-dialysis. Consistent with previous studies (Ravid and Chitalia, 2020), uremic solutes were enriched for products involved in amino acid, urea metabolism, and TCA pathways and other known markers of CKD (Supplementary Table S1). These included marked accumulation of creatinine (breakdown product of creatine normally cleared by the kidney (Zhang and Parikh, 2019)), asymmetric dimethylarginine (ADMA; an inhibitor of nitric oxide synthases linked to endothelial dysfunction), 4-hydroxyquinoline, acetylcarnitine, taurine, creatine, and homocysteine. (Figure 1D).

Strikingly, among all the significantly changed metabolites (1,167 increased and 186 decreased compounds, 1,353 in total) detected in ESKD as compared with the healthy cohort, only half (51.5%) of the elevated uremic compounds were quantitatively altered by hemodialysis (category ‘I’; Figure 1C). Of the remainder, 34.7% serum metabolites remained significantly elevated in ESKD even post-dialysis (Post, *green*) relative to baseline levels (category ‘II’), while 13.7% showed reduced levels compared with the healthy cohort (category ‘III’ in Figure 1C). The former included creatinine, whose levels in ESKD remained 7.5 times higher on average even post-dialysis than those in control (Figure 1D), as well as uric acid and 41 putative metabolites (FC > 5, *p* < 0.05; Supplementary Table S1) that remained unclear in previous CKD analyses (Liu et al., 2017; Ravid and Chitalia, 2020), such as cystine, 5-hydroxyindole-3-acetic acid (5-HIAA), 4-acetamidobutyric acid, indole-3-carboxaldehyde, 12-hydroxyeicosatetraenoic acid (12-HEPE), cadaverine, and linoleic acid (Figure 1E).

Interestingly, while most of the metabolites maintained high circulating levels in patient’s blood, we found that metabolites including tryptophan, dimethyglycine, phosphocreatine, and acetyl-tryptophan were significantly lower in ESKD than those in the healthy cohort. Some of these metabolites might play important roles in progression of CKD. As reported in a previous study with the large cohort (n = 1915), a lower level of plasma tryptophan and high kynurenine–tryptophan ratios were associated with a high risk for ESKD progression. The tryptophan–kynurenine pathway alteration in ESKD showed a strong correlation with type 2 diabetes (Liu et al., 2023), which is one of the commonly observed complications in ESKD patients. This result was consistent with our finding, as shown in Figure 1D (increased kynurenine and decreased tryptophan), suggesting that shunting the tryptophan catabolism in kynurenine may slow the CKD progression.

Although metabolite identification remains challenging, a stringent match score cut-off (>0.7) was used to establish high-confident database (MSDIAL) search results (see Figures 1D, E for several spectra matching cases). We independently validated 21 of these candidate metabolite markers using reference standards (MetaSCI) using both MS/MS and retention time matching (Table 2).

**TABLE 2:** Elevated metabolites identified in the serum from end-stage kidney disease patients confirmed by reference standards.

Metabolite name	Formula	m/z	Average Rt, min	Rt ∆,min	MS/MS matched	Adduct type	Accession
2,3-Dihydroxybenzoic acid	C_7_H_6_O_4_	153.01921	31.0	0.4	TRUE	[M-H]-	HMDB0000397
4-Hydroxyquinoline	C_9_H_7_NO	146.05943	18.6	−0.8	TRUE	[M + H]+	PUBCHEMCID69141
6-Methylcoumarin	C_10_H_8_O_2_	161.05898	24.9	−0.1	TRUE	[M + H]+	HMDB0032394
Caffeine	C_8_H_10_N_4_O_2_	195.08676	15.0	0.7	TRUE	[M + H]+	HMDB0001847
Creatine anhydrous	C_4_H_9_N_3_O_2_	132.07625	0.8	0.2	TRUE	[M + H]+	HMDB0000064
Creatinine	C_4_H_7_N_3_O	114.06605	0.4	0.5	TRUE	[M + H]+	HMDB0000562
Famotidine	C_8_H_15_N_7_O_2_S_3_	338.05096	10.3	1.5	TRUE	[M + H]+	HMDB0001919
L-Arginine	C_6_H_14_N_4_O_2_	175.1183	0.2	2.0	TRUE	[M + H]+	HMDB0000517
L-Glutamine	C_5_H_10_N_2_O_3_	147.07596	1.3	−0.4	TRUE	[M + H]+	HMDB0000641
L-Kynurenine	C_10_H_12_N_2_O_3_	209.09135	11.5	−0.9	TRUE	[M + H]+	HMDB0000684
L-Leucine	C_6_H_13_NO_2_	132.10136	4.5	−0.7	TRUE	[M + H]+	HMDB0000687
L-Phenylalanine	C_9_H_11_NO_2_	166.08556	10.4	0.3	TRUE	[M + H]+	HMDB0000159
L-Proline	C_5_H_9_NO_2_	116.07044	2.9	1.8	TRUE	[M + H]+	HMDB0000162
L-Tryptophan	C_11_H_12_N_2_O_2_	205.09622	12.3	0.2	TRUE	[M + H]+	HMDB0000929
L-Tyrosine	C_9_H_11_NO_3_	182.08064	4.3	0.7	TRUE	[M + H]+	HMDB0000158
LPC 16:0	C_24_H_50_NO_7_P	496.3385	35.5	0.5	TRUE	[M + H]+	HMDB0010382
Piperine	C_17_H_19_NO_3_	286.1423	31.3	−0.7	TRUE	[M + H]+	HMDB0029377
Riboflavin	C_17_H_20_N_4_O_6_	375.13385	15.5	0.1	TRUE	[M-H]-	HMDB0000244
Taurine	C_2_H_7_NO_3_S	124.00748	1.3	0.7	TRUE	[M-H]-	HMDB0000251
Theobromine	C_7_H_8_N_4_O_2_	181.07126	12.2	1.7	TRUE	[M + H]+	HMDB0002825
Uracil	C_4_H_4_N_2_O_2_	113.03434	7.5	−0.8	TRUE	[M + H]+	HMDB0000300

### Persistent elevation of lipids in ESKD patients undergoing hemodialysis

**FIGURE 2:**
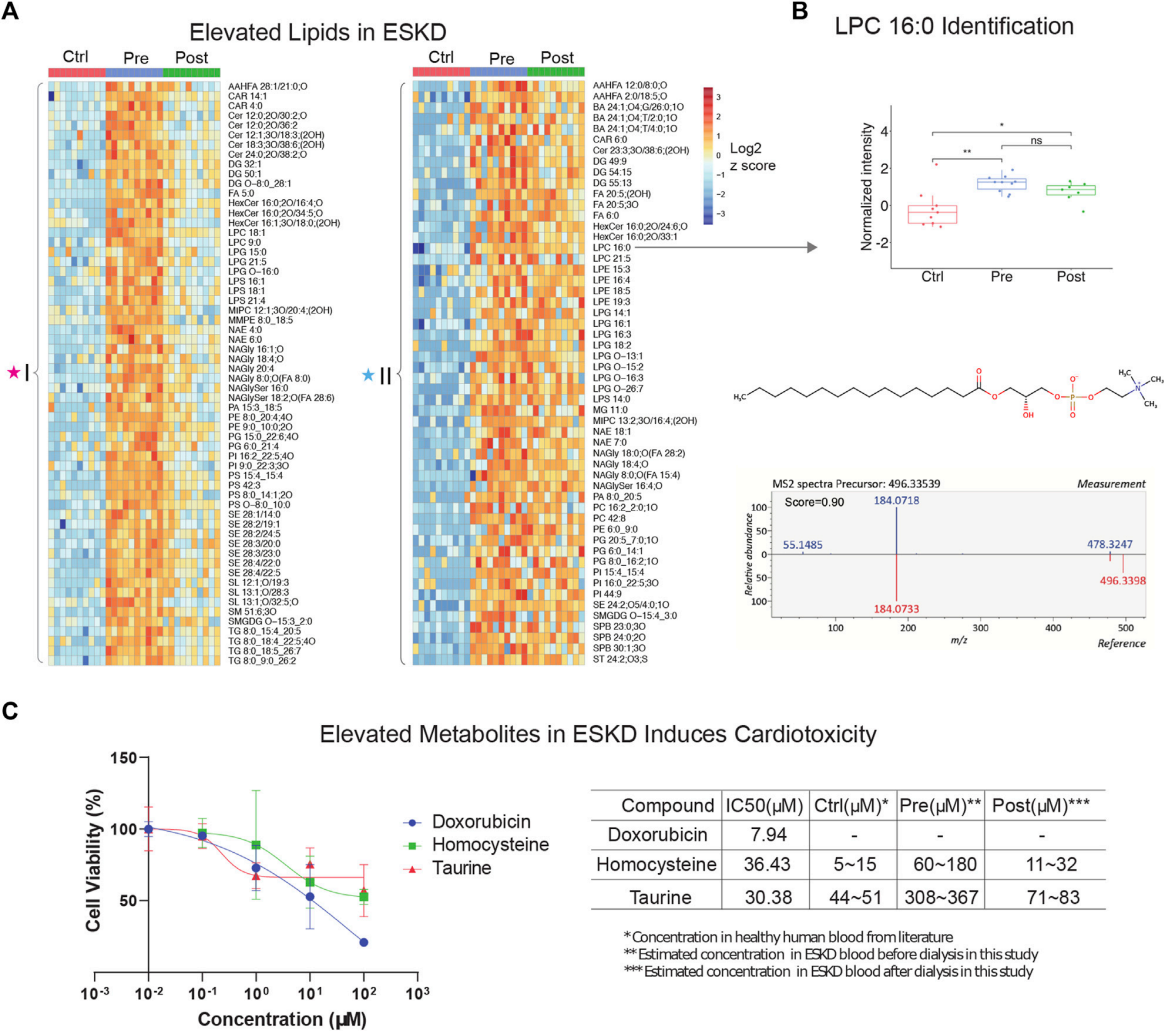
Untargeted lipidomic profiling and cardiotoxic compounds of patients with end-stage kidney disease **(A)** Heatmap of lipids significantly elevated (*p* < 0.05, >5-FC, FDR<0.05) in pre-dialysis patients compared with controls, as measured by PANAMA (LLE-SPME-nLC/MS). **(B)** Representative data showing the circulating levels and MS/MS fragmentation patterns recorded for lysoPC (16:0). **(C)** iPSC-derived cardiomyocyte viability response curves of positive control (doxorubicin) and ESKD-elevated uremic metabolites (homocysteine and taurine); accompanying table shows IC_50_ and estimated concentrations in circulation in ESKD pre- and post-hemodialysis patients and healthy controls (Ctrl).

Our metabolic profiling also revealed numerous circulating lipids, showing differential accumulation in ESKD (Supplementary Table S1) from the same nLC-MS run. Consistent with previous studies (Reis et al., 2015; Chen et al., 2017), five main lipid classes were significantly elevated in the pre-dialysis samples (Figure 2A): glycerophospholipids (PC, PE, and PG), glycerolipids (TG, DG, and MG), sphingolipids (Cer, MIPC, SM, and SPB), sterol lipids (SE, ST, and BA), lysophospholipids (LPC, LPE, and LPG), and fatty acyls (FA). For example, LysoPC 16:0 was elevated significantly (FC = 8.9, *p* < 0.01) in pre-dialysis and maintained a high concentration even after dialysis compared with Ctrl (FC = 6.6, *p* < 0.01) (Figure 2B). The identity of LysoPC 16:0 was confirmed by MS2 spectral analysis and retention time matching using a reference standard (Figure 2B; Table 2). Although ceramides (Cer), steryl esters (SE), and glycerolipids (TG) were largely cleared by dialysis (Figure 2A), we also found that bile acids and lysophospholipids, such as lysophosphatidylethanolamine (LPE), lysophosphatidylcholine (LPC), and lysophosphatidylglycerol (LPG), were not efficiently removed, potentially contributing to the high incidence of CVD observed in ESKD.

### Uremic toxins link to cardiovascular disease

To investigate the putative effect of persistent metabolites on cardiovascular disorders often observed in CKD, we performed a cytotoxicity assay with human iPSC-derived cardiomyocytes. Among the hundreds of elevated metabolites in ESKD compared with the healthy cohort, metabolites associated with methionine metabolism (e.g., methionine and homocysteine), glycine and serine metabolism, and taurine metabolism were identified. Homocysteine, a sulfur-containing amino acid derived from methionine metabolism, was previously reported to increase the risk of CVD using rodent cardiomyocytes isolated from mice or rats (Sipkens et al., 2007; Wang et al., 2012), which are imperfect models of human pathophysiology. In contrast, we examined the toxicity of these compounds using human iPSC-derived cardiomyocytes. Although, other non-myocyte cells, including smooth muscle cells, endothelial cells, cardiofibroblasts, and epicardium, have been broadly used in cardiac condition evaluation (Funakoshi and Yoshida, 2021), iPSC-derived cardiomyocytes are a widely used model that has been used extensively for cardiotoxicity screenings (Fonoudi and Burridge, 2021). This model faithfully recapitulates characteristics unique to human pathobiology and provides a less heterogeneous source of cells, which we feel offers a better validation system for evaluating the potential clinical relevance of metabolites we found at persistently high levels in the human serum. Likewise, taurine, a sulfur amino acid-like compound similar to methionine and homocysteine, has been linked to cardiovascular disease during impaired kidney function (Bkaily et al., 2020). Although it is difficult to make a conclusion about the beneficial and possible toxic effects of taurine, particularly the effect on cardiovascular under ESKD, reaching a clear understanding requires further exploration.

Here, we evaluated two selected metabolites, homocysteine and taurine, to determine their relative cytotoxicity effect using DOX (a known cardiotoxin) as a positive control. It has been demonstrated that iPSC-derived cardiomyocytes exposed to DOX show a decrease in cell viability, increased intracellular Ca^2+^, resulting in a decrease in spike amplitude, and an increase in the beat rate. These effects are correlated and are observed immediately after exposure and worsened with prolonged treatment (7–14 days) (Maillet et al., 2016). Notably, we observed homocysteine and taurine reduced cell viabilities (Figure 2C). In comparison with the IC_50_ value of doxorubicin (7.94 μM), a compound with known cytotoxic effects on cardiomyocytes, the IC_50_ values for homocysteine and taurine were 36.43 μM and 30.38 μM, respectively. In healthy human plasma, the concentration of homocysteine is typically 5–15 μM (Wang et al., 2019), whereas we estimate that the concentration of homocysteine in ESKD patients before dialysis is at least 12 times higher (60–180 μM, Figure 2C) than that in the control cohort using our precision LC-MS as relative quantification. The concentration was estimated using the relative intensities after normalization across control, and pre- and post-dialysis groups. The concentration of taurine was also seven times higher (estimated 308–367 μM) in the ESKD patient serum than that found in normal human plasma (Trautwein and Hayes, 1995) and remains 1.6 times higher even after hemodialysis. Significantly elevated blood concentrations of these compounds in patients potentially contribute to CVD and poor clinical outcomes. Unlike for DOX, there was no significantly altered contraction observed for any of the concentrations of homocysteine and taurine tested. Hence, future studies are required to assess long-term functional consequences on heart cell function.

### Parallel proteomic analysis reveals proteins elevated in ESKD

**FIGURE 3:**
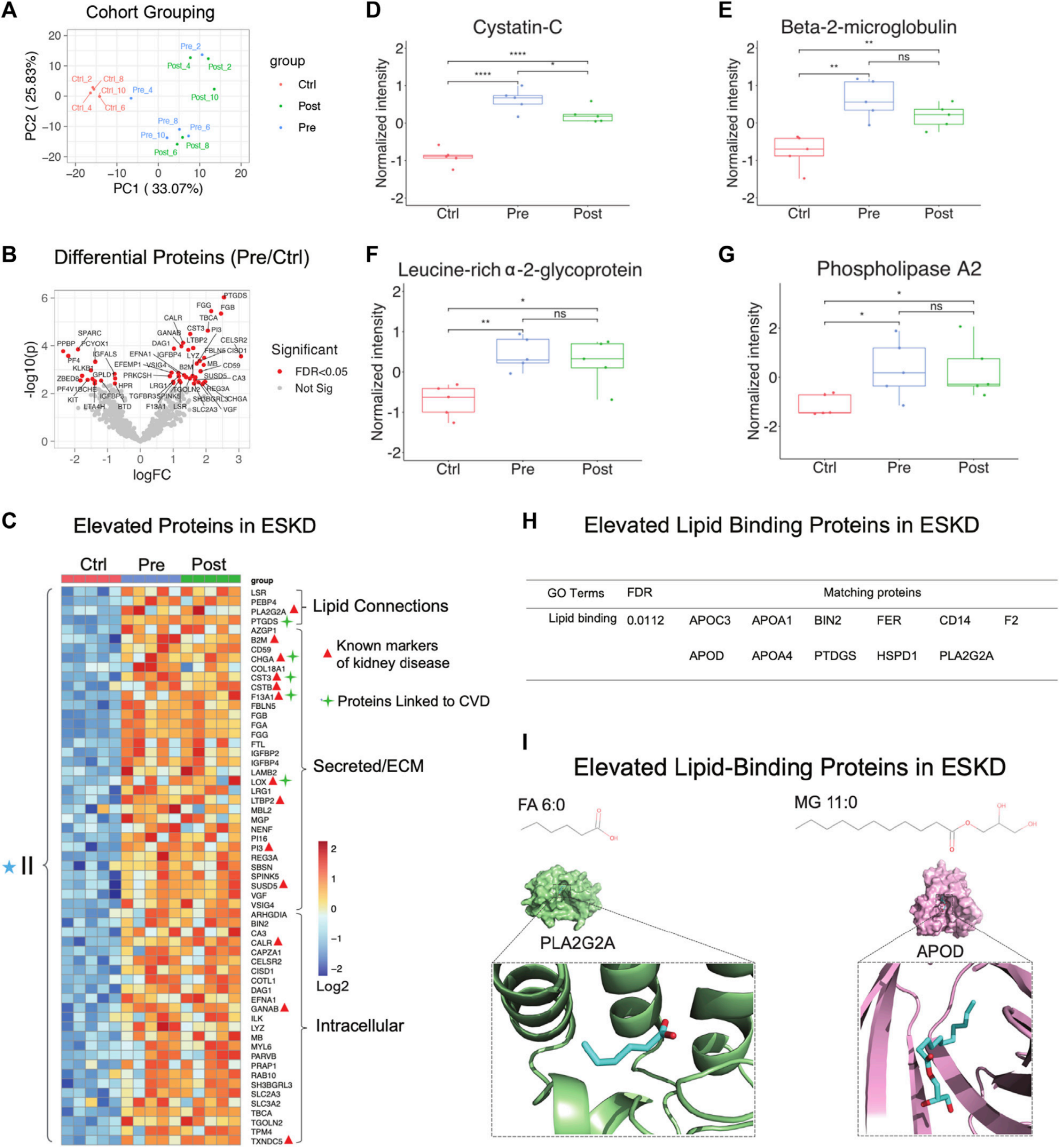
Parallel proteomic profiles of end-stage kidney disease. **(A)** PCA plot of blood sample profiles (healthy controls vs*.* pre- and post-dialysis ESKD cohorts). **(B)** Volcano plot of protein change in ESKD patients compared with controls (FDR <0.05). **(C)** Heatmap of persistently elevated proteins (*p* < 0.05, >2-FC, FDR<0.05) in pre-dialysis serum *versus* control samples; markers related to CKD and CVD are highlighted. **(D–G)** Relative circulating levels of cystatin c, beta-2-microglobulin, leucine-rich alpha-2-glycoprotein, and phospholipase A2 in the ESKD patient serum. **(H)** Significant elevated lipid-binding proteins in ESKD (FDR<0.05). **(I)** Lipid metabolite structures and protein–lipid docking models for phospholipase A2 (PLA2G2A; *green*) with FA (6:0) (*cyan*) and apolipoprotein D (APOD; *pink*) with MG 11:0 (*cyan*).

PCA showed that hemodialysis (using an Optiflux filter consisting of an inner diameter of ∼200 microns) did not significantly alter circulating protein levels (Figure 3A, post vs. pre). As expected, among all 83 proteins which were significantly elevated (*p* < 0.05, ≥1.5-fold-change) in ESKD patients relative to controls (Figure 3B, C), only 19 of them got slightly decreased after dialysis (1.2 fold-change in post vs. pre)**.** In addition to known markers of renal dysfunction that accumulate when glomerular filtration is impaired (George and Gounden, 2019), such as PTGDS, LTBP2, F13A1, and the secreted factors cystatin C (CST3, Figure 3D) and beta-2 microglobulin (B2M) (Figure 3E), we also detected leucine-rich alpha-2-glycoprotein (LRG1) (Figure 3F), previously suggested (Glorieux et al., 2015) as an indicator of vascular damage and heart failure; TXNDC5, a mediator of cardiac fibrosis (Chen et al., 2021); and calreticulin (CALR, Figure 3C), a marker of renal fibrosis (Lu et al., 2020).

Consistent with the large-scale change in circulating lipids we detected, elevated levels in multiple lipid-binding proteins were observed, such as phospholipase A2 (PLA2G2A, Figure 3G), lipolysis-stimulated lipoprotein receptor (LSR), phosphatidylethanolamine-binding protein 4 (PEBP4), prostaglandin-H2 D-isomerase (PTGDS), and apolipoprotein C3/D) (Figure 3H). Co-upregulated pairs include high-confidence associations (Alekseenko et al., 2020) between APOD and monoacylglycerol (MG, 11:0), and PLA2G2A with FA (6:0) (Figure 3I), reflecting a direct functional coupling between these proteins and their corresponding lipid ligands.

**FIGURE 4:**
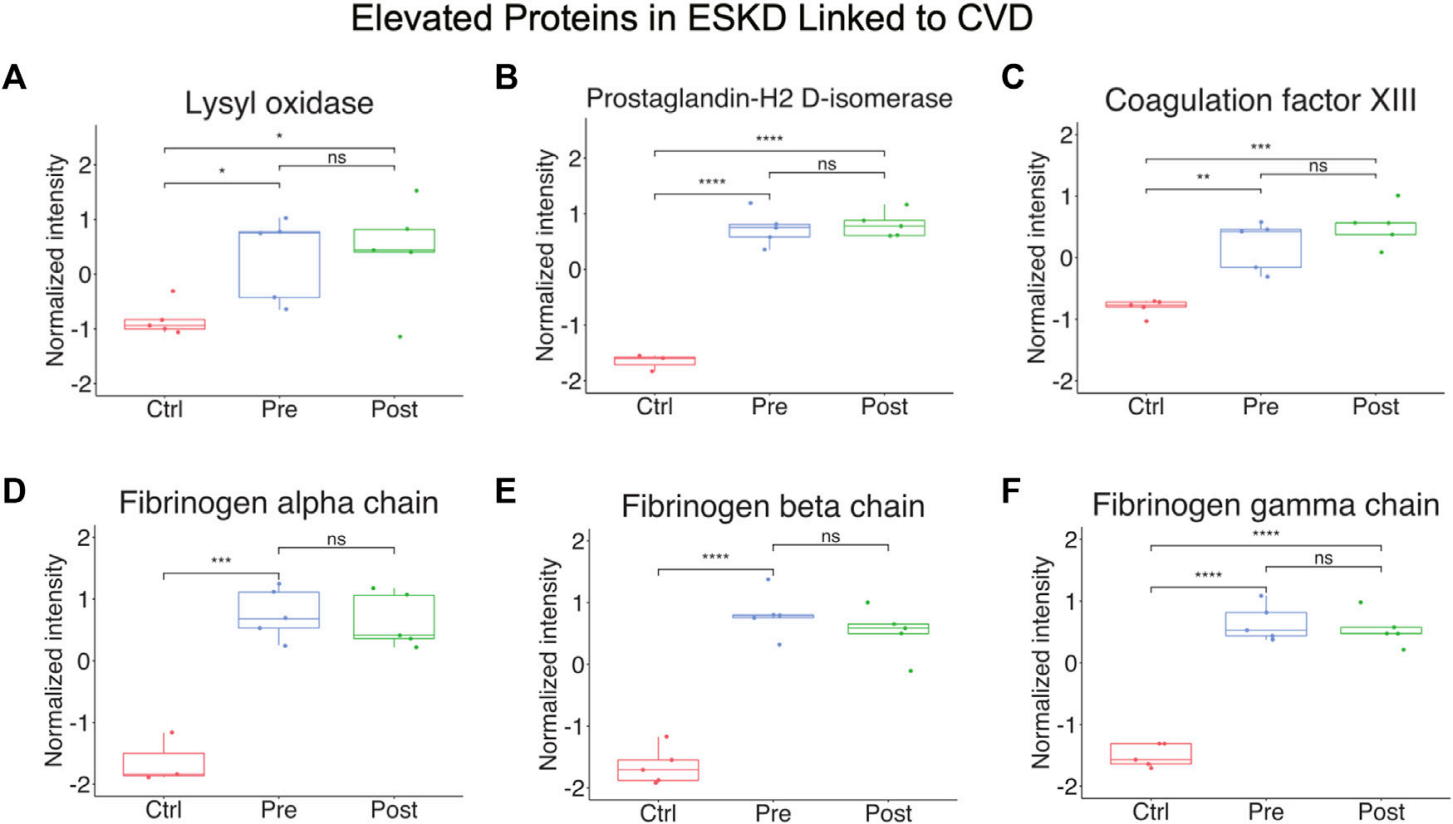
Elevated proteins in ESKD linked to CVD. **(A–F)** Proteins persistently elevated in ESKD linked to CVD include lysyl oxidase, prostaglandin-H2 D-isomerase, coagulation factor XIII, and fibrinogen.

Other notable alterations in ESKD included upregulation of factors involved in cell adhesion, fibrosis, and blood coagulation (Figure 4A-F), and the downregulation of inhibitors of inflammation. The former included integrin-linked protein kinase (ILK), implicated in cellular dysfunction, fibrosis, and inflammation in animal studies (Zhao et al., 2015), and lysyl oxidase (LOX, Figure 4A), an extracellular enzyme known to control fibrosis (Papadantonakis et al., 2012), while the latter included factors associated with atherothrombosis, such as PTGDS (platelet aggregation, Figure 4B), F13A1 (subunit of plasma coagulation factor XIII associated with arterial and venous thrombosis (Carreras-Torres et al., 2010)), and fibrinogen (Figure 4D-F), which is associated with recurrent DVT and pulmonary embolism (Bereczky et al., 2007)). These findings are particularly noteworthy, given that these clinical events had a high occurrence in our EKSD patient cohort (Table 1).

### Dual workflow provides an integrated protein–metabolite network associated with ESKD

**FIGURE 5:**
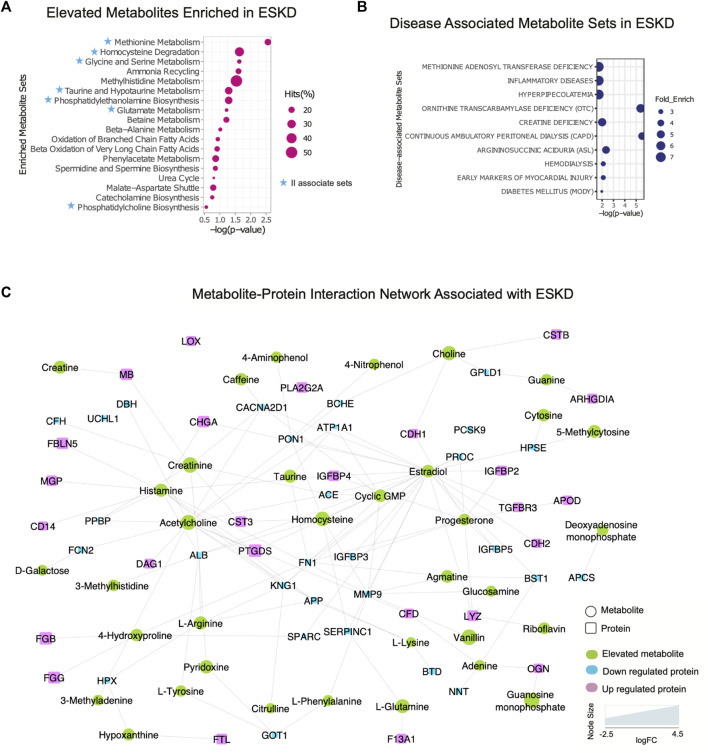
Integrated protein–metabolite network reveals biochemical interactions associated with ESKD. **(A, B)** Results of the annotated pathway and disease-associated protein enrichment analysis showing significantly altered abundance (*p* < 0.05, >2-FC, categories I and II) in the ESKD patient serum. **(C)** Metabolite–protein (circle and square shapes, respectively) interaction network associated with ESKD.

Metabolomic pathways play an important role in waste product breakdown and excretion in ESKD such that a better understanding of these pathways is essential to improve patient outcomes. To identify pathways and biological processes in which these metabolites played a role, we performed enrichment analysis using the Small Molecule Pathway Database (SMPDB). As shown in Figure 5A, elevated metabolites (categories I and II) associated with ESKD were involved in diverse metabolomic pathways, including ammonia recycling and urea cycle, and when the kidney failed to convert urea and ammonia into less toxic form (Weiner et al., 2015), along with methionine metabolism due to the poor renal clearance and degradation of homocysteine, a byproduct of methionine, they were not efficiently removed by dialysis (Zhloba and Subbotina, 2022), as well as oxidation of fatty acids and amino acid metabolism (glycine, serine, methylhistidine and glutamate metabolism, etc.) (Figure 5A) (van de Poll et al., 2004). Additionally, compounds linked to taurine metabolism, phosphatidylethanolamine biosynthesis, betaine metabolism, phenylacetate metabolism, and phosphatidylcholine biosynthesis were found to be elevated in the blood of ESKD patients. Among these, metabolites related to ammonia recycling, urea cycle, methylhistidine metabolism, and phenylacetate metabolism were efficiently removed by dialysis (FC > 2, *p* < 0.05 of post/pre), whereas uremic compounds involved in methionine metabolism, homocysteine degradation, glycine and serine metabolism, taurine and hypotaurine metabolism, phosphatidylethanolamine biosynthesis, glutamate metabolism, and phosphatidylcholine biosynthesis remained elevated after hemodialysis (Figure 5A, *blue star*). Since persistent compounds are more likely to be associated with the poor outcomes, we tested some candidates’ adverse effects *in vitro* and confirmed that homocysteine and taurine are cardiotoxic (Figure 2C).

We extended our investigation to explore the correlation of the elevated metabolites in the ESKD patient and clinical disease. Our disease enrichment analysis (Figure 5B) revealed metabolites (categories I and II) related to creatine deficiency and hemodialysis along with methionine adenosyl transferase, hyperpipecolatemia, and ornithine transcarbamylase deficiency, which depend on kidney clearance, and compounds linked to ESKD complications such as inflammation, diabetes (energy metabolism disturbance), and early markers on myocardial injury (presumably due to a larger blood volume that increases the heart workload in CKD) (Aoki and Ikari, 2017).

To explore the potential association between metabolites and proteins, we searched the STITCH database (Kuhn et al., 2008), which considers factors such as the chemical structure and molecular activities to establish physical or functional connections between small-molecule compounds and polypeptides. As shown in Figure 5C, creatinine, a key biomarker of CKD, showed direct physical interactions with the blood proteins CST3, MB, serum albumin (ALB), angiotensin-converting enzyme (ACE), complement factor H(CFH), and voltage-dependent calcium channel subunit alpha-2/delta-1 (CACNA2D1), while homocysteine was connected to CST3, serum paraoxonase/arylesterase 1 (PON1), and antithrombin-III (SERPINC1) (Figure 5C). Both increased creatinine and cystatin C (CST3) are commonly measured to estimate the kidney function. Clinical studies have demonstrated that a high creatinine–cystatin C ratio is correlated with better survival of CKD, which can reflect changes in muscle mass (Jung et al., 2021). For high creatinine levels, lowering blood cystatin C levels could be a strategy to potentially lower mortality. Conversely, the decrease in serum albumin (indirect indication of high urine ALB) associated with a decline renal function was apparent (Figure 5C), consistent with the albumin-to-creatinine ratio as a risk factor for diabetes-related outcomes (Gerstein et al., 2022). Importantly, the serum protein–metabolite interactions that annotated in our integrated analysis revealed a larger network encompassing other components associated with clinical complications of ESKD. For instance, meta-analysis revealed that serum paraoxonase 1 (PON1) activity is likely reduced in CKD (decreased level shown in Figure 5C), suggesting the antioxidant defense is impaired (Watanabe et al., 2023). The beneficial role of PON1 activity on reducing the cardiovascular event has been reported (Shih and Lusis, 2009). Intriguingly, the interaction of homocysteine and PON1 implies that changes in their levels exert a synergistic effect on clinical outcomes in ESKD patients.

Moreover, interactions/bindings of ALB with many other metabolites including creatinine, homocysteine, uric acid, arginine, pyridoxine, tyrosine, and 3-methylhistidine might relate to the low filtering efficiency for small molecules during dialysis (Figure 5C). A comprehensive protein network was annotated including the interactions of ESKD protein biomarkers (PTGDS, F13A1, and CSTB) with acetylcholine, histamine, choline, and lipid-binding proteins (PLA2G2A, PTGDS, APOD, and MG) with linoleic acid, isovaleric acid, atorvastatin, creatine, creatinine, and enzyme related to fibrinogen (LOX) with linoleic acid (Figure 5C). This global serum protein–metabolite interaction network enables exploration and visualization of functionally-related (through physical interactions) metabolites and proteins associated with ESKD and associated clinical outcomes.

## Discussion

The identity of chronically dysregulated metabolites, lipids, and proteins in the blood of patients being treated for ESKD is not fully known (Slocum et al., 2012). To this end, we applied dual convergent metabolomics and proteomic workflows to characterize circulating biomolecules that remain elevated despite hemodialysis. This may reflect binding to blood proteins (*e.g*., albumin) that precludes clearance by filtration. Persistently elevated levels of uremic compounds have been implicated in serious clinical complications, including CVD (Ravid et al., 2021), as well as bone and mineral disruption, anemia, and infertility in patients with CKD (Zalunardo and Levin, 2006; Raggi and Kleerekoper, 2008; Dumanski and Ahmed, 2019), but the products that are differentially retained in CKD remain incompletely characterized.

Our cytotoxicity assay demonstrated that two of the identified uremic compounds, homocysteine and taurine, significantly reduced human iPSC-derived cardiomyocyte viability, which is a well-established model to determine cardiotoxicity (Satsuka and Kanda, 2020). Elevated homocysteine has previously been implicated in cardiac remodeling and coronary heart disease (Kar et al., 2019), while taurine has been proposed to have a cardioprotective effect (Takatani et al., 2004). Although our taurine results are counterintuitive, prior studies documented its anti-apoptotic effect in the context of ischemia or heart disease. Although further investigation is required to determine their molecular targets in cardiac cells, our preliminary results are consistent with a putative role for these persistently elevated compounds in the observed cardiac phenotypes commonly observed in CKD.

High levels of primary bile acids have been reported to contribute to cardiac hypertrophy and heart failure (Desai et al., 2017), common risk factors for mortality associated with dialysis. The activation of PLA2 leads to the release of FA and elevation of the LPC level. Chronic elevation of these markers may be one reason for the accelerated atherosclerosis and CVD-related complications commonly observed in ESKD (Nusair et al., 2012). In CVDs, LPC induces macrophages to uptake oxidized low-density lipoproteins (ox-LDLs), leading to the formation of foam cells, and aggravates the development of atherosclerotic plaques (Aiyar et al., 2007; Reis et al., 2015), which is linked to the high incidence of CVD in dialysis patients. LPC’s effects on endothelial cells and vascular smooth muscle cells play a vital role in the progression on CVDs (Liu et al., 2020). However, further studies are needed to investigate the effects of potential biomarkers, including LPC, PLA2, homocysteine, taurine, and other persistent metabolites we have identified in various disease models independently (e.g., endothelial cell and smooth muscle cells). It has been demonstrated that LPC, particularly 16:0, increases the release of inflammatory cytokines from endothelial cells (Morita et al., 2015). Additionally, LPC promotes the expression of adhesive molecules and enhances the adhesive effect of endothelial cells. Therefore, further evaluation of these effects of uremic toxins would shed light on the mechanism of the correlation between ESKD and heart failure in patients.

In summary, integration of untargeted metabolomics and proteomics enabled the exploration of multiple analyte types and their associated interactions starting from limiting amounts of biospecimens rich in interfering matrix components using widely available instrumentation to identify potentially clinically actionable prognostic markers of disease progression. Our integrated metabolite–protein network generates a comprehensive picture of the ‘disease interactome’ associated with ESKD patients despite them undergoing hemodialysis, providing mechanistic insights for better clinical management.

## Data Availability

The datasets presented in this study can be found in online repositories. The names of the repository/repositories and accession number(s) can be found in the article/Supplementary Material. Data for this project have been deposited to the MassIVE archive accession code MSV000091579 (doi:10.25345/C5CV4C23Z).
