# Corrigendum: Integrated metabolomics and proteomics reveal biomarkers associated with hemodialysis in end-stage kidney disease

**DOI:** 10.3389/fphar.2024.1376058

**Published:** 2024-02-09

**Authors:** Weiwei Lin, Fatemeh Mousavi, Benjamin C. Blum, Christian F Heckendorf, Jarrod Moore, Noah Lampl, Mark McComb, Sergei Kotelnikov, Wenqing Yin, Nabil Rabhi, Matthew D. Layne, Dima Kozakov, Vipul C. Chitalia, Andrew Emili

**Affiliations:** ^1^ Center for Network Systems Biology, Boston University, Boston, MA, United States; ^2^ Department of Biochemistry, Boston University School of Medicine, Boston, MA, United States; ^3^ Department of Applied Mathematics and Statistics, Stony Brook University, Stony Brook, NY, United States; ^4^ Renal Section, Department of Medicine, Boston University School of Medicine, Boston, MA, United States; ^5^ Veterans Affairs Boston Healthcare System, Boston, MA, United States; ^6^ Institute of Medical Engineering and Sciences, Massachusetts Institute of Technology, Cambridge, MA, United States; ^7^ Department of Biology, Boston University, Boston, MA, United States

**Keywords:** metabolomics, proteomics, nLC-MS/MS, ESKD, integrated omics

In the published article, there was an error in the **Funding** statement. The current statement is “This work was supported by operating funds to AE from the Canadian Institutes of Health Research (FDN-148399) and generous start-up funds from Boston University.”

The correct **Funding** statement appears below.

“This work was supported by operating funds to AE from the Canadian Institutes of Health Research (FDN-148399) and generous start-up funds from Boston University. This work was also supported by funds to VC from the National Heart Lung and Blood Institute (1R01HL166608).”

The authors apologize for this error and state that this does not change the scientific conclusions of the article in any way. The original article has been updated.

