# Supplementary material for: Integrated metabolomics and proteomics reveal biomarkers associated with hemodialysis in end-stage kidney disease
Source: Front Pharmacol. 2023 Nov 27;14:1243505. doi: 10.3389/fphar.2023.1243505 (PMC10715419; doi:10.3389/fphar.2023.1243505)
Supplement: Supplementary file 1 [file Table1.DOCX]

**Table 1. Patient characteristics**

| **Study/baseline participant** | **Patients with ESKD on HD** | **Controls** |
| --- | --- | --- |
|  | **N= 10** | **N = 10** |
| Age, year# | Mean: 64.4 + 10.22 | Mean: 50.2 + 8.61 |
| Male | 5 (50%) | 6(60%) |
| BMI # | Mean: 31.63 + 5.39 | Mean: 25.72 + 7.25 |
| Ethnicity |  |  |
| Hispanic | 2 | 6 |
| Non-Hispanic | 8 | 4 |
| Race |  |  |
| Blacks | 8 | 4 |
| Whites | 2 | 6 |
| Asians | 0 | 0 |
| American Indians | 0 | 0 |
| Others | 0 | 0 |
| Smoke | 6 | 0 |
| Hypertension | 10 | 0 |
| Diabetes Mellitus | 8 | 0 |
| Cardiovascular diseases |  |  |
| Documented CAD | 5 | 0 |
| Angina | 6 | 0 |
| Coronary Artery Bypass | 5 | 0 |
| Congestive Heart Failure | 5 | 0 |
| hypercholesterolemia | 5 | 0 |
| Peripheral vascular diease | 2 | 0 |
| Deep Venous thrombosis | 6 | 0 |
| Pulmonary embolism | 2 | 0 |
| Blood pressure, mm Hg |  |  |
| Systolic# | Mean: 140.7 + 22.31 | Mean: 125 + 14.69 |
| Diastolic# | Mean: 79.2 + 15.89 | Mean: 74 + 6.53 |
| eGFR ml/min/sq mt Body | Mean: 8.1 + 7.89 | Mean: > 90 |
