# Supplementary material for: Integrated metabolomics and proteomics reveal biomarkers associated with hemodialysis in end-stage kidney disease
Source: Front Pharmacol. 2023 Nov 27;14:1243505. doi: 10.3389/fphar.2023.1243505 (PMC10715419; doi:10.3389/fphar.2023.1243505)
Supplement: Supplementary file 2 [file Table2.DOCX]

**Table 2. Elevated metabolites identified in serum from end-stage kidney disease patients confirmed by reference standards**

| Metabolite Name | Formula | m/z | Average Rt,min | Rt ∆,min | MS/MS matched | Adduct type | Accession |
| --- | --- | --- | --- | --- | --- | --- | --- |
| 2,3-Dihydroxybenzoic acid | C7H6O4 | 153.01921 | 31.0 | 0.4 | TRUE | [M-H]- | HMDB0000397 |
| 4-Hydroxyquinoline | C9H7NO | 146.05943 | 18.6 | -0.8 | TRUE | [M+H]+ | PUBCHEMCID69141 |
| 6-Methylcoumarin | C10H8O2 | 161.05898 | 24.9 | -0.1 | TRUE | [M+H]+ | HMDB0032394 |
| Caffeine | C8H10N4O2 | 195.08676 | 15.0 | 0.7 | TRUE | [M+H]+ | HMDB0001847 |
| Creatine anhydrous | C4H9N3O2 | 132.07625 | 0.8 | 0.2 | TRUE | [M+H]+ | HMDB0000064 |
| Creatinine | C4H7N3O | 114.06605 | 0.4 | 0.5 | TRUE | [M+H]+ | HMDB0000562 |
| Famotidine | C8H15N7O2S3 | 338.05096 | 10.3 | 1.5 | TRUE | [M+H]+ | HMDB0001919 |
| L-Arginine | C6H14N4O2 | 175.1183 | 0.2 | 2.0 | TRUE | [M+H]+ | HMDB0000517 |
| L-Glutamine | C5H10N2O3 | 147.07596 | 1.3 | -0.4 | TRUE | [M+H]+ | HMDB0000641 |
| L-Kynurenine | C10H12N2O3 | 209.09135 | 11.5 | -0.9 | TRUE | [M+H]+ | HMDB0000684 |
| L-Leucine | C6H13NO2 | 132.10136 | 4.5 | -0.7 | TRUE | [M+H]+ | HMDB0000687 |
| L-Phenylalanine | C9H11NO2 | 166.08556 | 10.4 | 0.3 | TRUE | [M+H]+ | HMDB0000159 |
| L-Proline | C5H9NO2 | 116.07044 | 2.9 | 1.8 | TRUE | [M+H]+ | HMDB0000162 |
| L-Tryptophan | C11H12N2O2 | 205.09622 | 12.3 | 0.2 | TRUE | [M+H]+ | HMDB0000929 |
| L-Tyrosine | C9H11NO3 | 182.08064 | 4.3 | 0.7 | TRUE | [M+H]+ | HMDB0000158 |
| LPC 16:0 | C24H50NO7P | 496.3385 | 35.5 | 0.5 | TRUE | [M+H]+ | HMDB0010382 |
| Piperine | C17H19NO3 | 286.1423 | 31.3 | -0.7 | TRUE | [M+H]+ | HMDB0029377 |
| Riboflavin | C17H20N4O6 | 375.13385 | 15.5 | 0.1 | TRUE | [M-H]- | HMDB0000244 |
| Taurine | C2H7NO3S | 124.00748 | 1.3 | 0.7 | TRUE | [M-H]- | HMDB0000251 |
| Theobromine | C7H8N4O2 | 181.07126 | 12.2 | 1.7 | TRUE | [M+H]+ | HMDB0002825 |
| Uracil | C4H4N2O2 | 113.03434 | 7.5 | -0.8 | TRUE | [M+H]+ | HMDB0000300 |
